# Disclosure following a medical error: lessons learned from a national initiative of workshops with patients, healthcare teams, and executives

**DOI:** 10.1186/s13584-024-00599-8

**Published:** 2024-03-11

**Authors:** Adi Finkelstein, Mayer Brezis, Amiad Taub, Dana Arad

**Affiliations:** 1 Department of Nursing, Faculty of Life and Health Sciences, Jerusalem College of Technology (JCT), Beit Hadfus 7 St., 9548307 Jerusalem, Israel; 2 School of Public Health, Hebrew University Hadassah Medical Center, Ein-Kerem, PO Box 12000, 91120 Jerusalem, Israel; 3 ‘Ofek Back to Life’ Organization, Sderot H’areches 13, 7178441 Modi’in, Israel; 4 Patient Safety Division, Ministry of Health, 39 Yeremiahu, 9101002 Jerusalem, Israel

**Keywords:** Dialogue, Disclosure, Healthcare providers, Israel, Listening, Medical malpractice, Medical error, Transparency

## Abstract

**Background:**

Despite the increase in disclosures of medical errors, transparency remains a challenge. Recognized barriers include shame, fear of litigation, disciplinary actions, and loss of patient trust. In 2018, the Israeli Ministry of Health initiated a series of workshops about disclosure of medical errors. The workshops involved medical center executives, healthcare providers, patients, and family members of patients who had previously been harmed by a medical error. This study presents the lessons learned about perceived challenges in disclosure of errors in 15 such workshops.

**Methods:**

Data collection included participant observations in 15 workshops, full audio recordings of all of the workshops, and documentation of detailed field notes. Analysis was performed under thematic analysis guidelines.

**Results:**

We identified four main themes: “Providers agree on the value of disclosure of a medical error to the patient”; “Emotional challenges of disclosure of medical error to patients”; “The medico-legal discourse challenges transparency”; and “Providers and patients call for a change in the culture regarding disclosure of medical errors”. Participant observations indicated that the presence of a patient who had experienced a tragedy in another hospital, and who was willing to share it created an intimate atmosphere that enabled an open conversation between parties.

**Conclusion:**

The study shows the moral, human, and educational values of open discourse in a protective setting after the occurrence of a medical error. We believe that workshops like these may help foster a culture of institutional disclosure following medical errors. We recommend that the Ministry of Health extend such workshops to all healthcare facilities, establish guidelines and mandate training for skills in disclosure for all providers.

## Background

Medical disclosure of errors refers to “communication between a healthcare provider and a patient, family member, or proxy that acknowledges the occurrence of an error, discusses what happened, and describes the link between the error and outcomes in terms the patient understands” [1]. There is widespread agreement that healthcare providers should disclose errors to patients and families [2–4].

Previous studies have acknowledged barriers to disclosure among providers: blame and shame [5, 6], fear of lawsuit or punishment, loss of patient trust, and inexperience communicating such information [2, 7, 8]. Other concerns include determining whether disclosure is necessary, when and how to disclose, who should disclose, and whether other team members, including risk management, should be present during disclosure [9].

Benefits of disclosure of errors by providers have been described. According to Toffolutti and Stuckler [10] evidence supports enhancing efforts to increase openness, transparency, and accountability across the hospital system, since doing so improves health care quality. A culture of openness is associated with a reduction in hospital mortality rates [10]. Disclosure has a healing effect psychologically [3]. It can ease patients’ pain without increasing litigation, and it facilitates actions to prevent recurrence. John [11] concluded that: “We need to work towards a different culture, one in which we openly acknowledge our own mistakes and that avoiding them completely is impossible”.

The benefits of disclosure justify overcoming the barriers to its implementation. In the United State, the Agency for Healthcare Research and Quality has suggested organizational tools for such disclosure [12] and the U.S. Joint Commission has linked this requirement to hospital accreditation [4]. Communication and resolution programs (CRPs) aim to enhance communication about events that did not involve negligence and to promote responsibility and transparency [13]. The objective of these programs is to provide immediate empathic responses to patient harm and implement lessons learned into safety improvements to prevent recurrences of the event [14]. Implementing CRPs may improve some liability outcomes [15, 16].

There is paucity of information about the disclosure of a medical error in Israel, and about the prevailing attitudes of health care providers and administrators toward disclosure in general. A report prepared by the Research and Information Center of the Parliament (“Knesset”) [17] showed that the Ministry of Health in Israel does not have comprehensive data on medical errors. Financial compensation paid for negligence claims in governmental medical institutions is often settled outside the courtroom, and details of these agreements remain confidential. Because of a lack of transparency, very little is known about the extent of medical errors in the healthcare system in Israel, their nature or severity, and what measures, if any, have been taken to prevent recurrence.

The aim of this paper was to explore attitudes of healthcare teams and executives to the challenge of disclosure as expressed at workshops with patients in a national initiative in Israel.

## Methods

### Settings

The workshops described in this article, occurred under the auspices of the Israel Ministry of Health in collaboration with Ofek-Back to Life [18], a non-profit organization. Ofek-back to Life, which is active in legislation and public activity, was established to support patients and families following a medical error and works to prevent such errors in the health care system. The workshops were organized to foster authentic human interaction between stakeholders rather than the typical adversarial exchanges of the courtroom. Prior experience with such face-to-face communication demonstrated the potential of the workshops for generating genuine communication with constructive ideas [19–21].

All 15 workshops were designed as 1-day meetings comprised of three sessions of about 60 min each. The first two sessions were structured: In the first session, there was an introduction with greetings from a representative of the host medical center, which was followed by a lecture delivered by the workshop facilitator (the second co-author), a senior physician and former director of the Center for Clinical Quality and Safety of Hadassah-Hebrew University Medical Center. Hadassah is a large tertiary hospital with rich research experience in the field of medical errors and disclosure [22, 23]. The lecture described the healing value of apology, transparency, and listening to restore truth. Short clips sampled from TED talks on errors and vulnerability were shown [20, 24, 25]. In the second session, a patient or a family member from *Ofek Back to Life* who had experienced a medical error *in another hospital* was invited to share their story without revealing any identifying details about the case itself. Then, the third session consisted of an open discussion guided by the facilitator. The discussion was organized as a panel that included the patient and/or family members that participated in the second session, senior management representatives of the hosting hospital (hospital’s manager or his/her deputy, risk manager, legal consultant) and a representative from the Ministry of Health. The declared task of the third session was to develop an open discussion between the physicians and the nurses who participated in the workshop and the panel members about the challenges of disclosure following the first and the second sessions of the workshop. The facilitator initiated the discussion asking the panelists to introduce themselves and inviting comments or questions from the audience.

### Data collection

#### Study population

It was a self-selected convenience sample as participation was voluntary. The workshops were offered to all hospitals and HMOs in the country and were open to physicians, nurses, and social workers. The invitation stressed a preference for those who held an executive position in addition to their clinical task (e.g., department chief, head nurse). The medical center distributed invitations by email to the members of the institution's health care team asking them to participate in a 1-day workshop dealing with the issue of disclosure following errors. The workshops were held over the course of 2 years (2/2018–1/2020) in 14 of the 29 hospitals (12 general hospitals and two geriatric hospitals) and one nursing school (Table 1).

**Table 1:** Workshop characteristics

Characteristics of workshops	N = 15 (%)
*Type*	
General hospital	12 (80)
Geriatric	2 (13.3)
Nursing school	1 (6.6)
*Hospital size*	
Large (> 800 beds)	3 (20)
Medium (400–800 beds)	3 (20)
Small (< 400 beds)	8 (53.3)
N/A	1 (6.6)
*Location*	
Urban	10 (66.6)
Rural	5 (33.3)
*Size of workshops*	
< 50 participants	1 (6.6)
50–100 participants	10 (66.6)
> 100 participants	4 (26.6)

Each workshop lasted an average of 3 h and most of the attendees were nurses and physicians (Table 2). An anonymous survey about attitudes towards disclosure was distributed to participants before the beginning of the workshop, and a second survey was distributed at the end of the workshop to rate its perceived value.

**Table 2:** Characteristics of the workshop participants

Participants’ characteristics	N = 997 (%)
*Gender*	
Male	337 (33.8)
Female	660 (66.1)
*Profession*	
Physicians	395 (39.6)
Nurses	512 (51.3)
Others	90 (9)
Executive role in addition to clinical tasks (e.g., department chief, head nurse)	850 (85.2)

#### Fieldwork

The fieldwork was conducted by the first co-author. Data collection included unstructured participant observation in 15 workshops followed by documentation of detailed field notes and audio recording of the workshops. Observation recording and field diary documentation continued during breaks and after the conclusion of the workshop until the last participant left. Unstructured observation is a method within the constructivist paradigm. Researcher usually enter the field with no set or prearranged notions as to what they might observe [26].

Participants in the workshops were fully informed about the observer’s (the first co-author) presence and the observation recordings for research purposes. Participants were informed that there was no need to mention their name, profession, or department and, in any case, all identifying details would be removed from the transcripts. Participants could request not to appear in the transcript. Only one participant made such a request.

### Data analysis

Only the third session of the 15 workshops was transcribed since it included the liveliest discussions. We removed the personal details of all participants to ensure anonymity. When participants identified themselves as nurses or physicians, we kept the information in the transcript. We assigned a random number of between 1 and 15 (hereafter: W1–W15) to each of the 15 workshops.

The verbatim transcripts were read and coded for emergent themes according to thematic analysis [27]. The first co-author, who is experienced in qualitative methodology conducted the analysis manually without the use of any software [28]. The other co-authors read the drafts of the analysis and gave ongoing feedback [29]. The final report was presented at a meeting at the Ministry of Health and at another meeting of risk managers from across the country.

## Results

Four main themes emerged from the qualitative analysis:

Theme 1: Providers agree on the value of disclosure of a medical error to the patient.

Providers agreed that transparency after the occurrence of a medical error is a critical component of the patient-physician relationship regardless of the outcome. They considered it to be a moral and professional value of restoring the patient’s and his/her family trust: “It is proper that families know what happened to them, or to their loved ones; it’s an important value” (W5); “… as soon as I shared that with the patient, I felt that I had at least closed a circle for myself and for her” (W1).

They mentioned honesty, sensitivity, and taking responsibility when talking to the patient: “Sometimes looking into their eyes and being there with them, even if you don’t say anything… … giving them the feeling that they are in good hands” (W1).

In a few cases, patients welcomed disclosure. “The father of that girl came to see me and said, ‘I’m asking you not to take any action against whoever made the error…promise me’” (W6). Others mentioned that disclosure provoked anger, which they were able to understand under the circumstances.

The importance of transparency with colleagues within the hospital and with colleagues from other hospitals was considered an essential part of medical professionalism to ensure future learning: “Information sharing is important. It can be done anonymously; it doesn’t matter to me what happened in which hospital… [what is important is] how I can prevent myself from falling into the same trap” (W6).

Theme 2: Emotional challenges of disclosure of medical error to patients.

- Physicians and patients acknowledged each other’s feelings.

In the observations, it was evident that the presence of one patient who had been harmed by a medical error in another hospital had an impact on what providers said and how they expressed themselves. The patient did not accuse anyone. Accordingly, the providers did not react defensively, which facilitated an authentic and sometimes emotional dialogue.

After the patient session, providers often started their remarks by expressing genuine sympathy to the patient. Some identified with the patient’s position:

Some day in the future, we and our families will return to the system as patients. I’m sorry to hear about the tragic case. We want to restore trust in us as a care team. … We need to develop a culture of acknowledging our mistakes and learning from them, which is the most important thing (Physician to the patient, W15).

During the breaks, physicians and nurses from the audience approached the patient, hugged them, thanked them. Some cried. In the closing panel, some of medical staff thanked the patients for sharing their personal stories and appreciated the opportunity to learn from the patients’ stories: “… You gave us something to think about. We hope that eventually patients will be treated with transparency. Your disclosure was moving” (Physician to the patient, W12); “It isn’t easy. It is not black and white … we are enthusiastic and willing to improve, to learn, and to cooperate. Thank you for coming. It was important” (Physician to the patient, W14).

The patients appreciated the opportunity to tell their stories to the medical center staff. They acknowledged the challenges faced by providers and addressed these challenges with understanding. “Thank you for the challenging questions. It showed me that it was important for you to learn” (Patient to the physician, W3); "First, I would like to thank you very much… it gives me strength to see that people who work in this profession care… You chose a tough job" (Patient, W7).

- Physicians and nurses shared their emotional difficulties following a medical error.

The physicians and nurses shared their feelings of guilt after a medical error occurred. “… The punishment is what one experiences through soul searching” (W4). They shared the shame and stigma following an admission of a medical error. They expressed their worry from the social and institutional reaction, which they thought might be an obstacle for revealing an error. “If someone makes a mistake, that person is not remembered for the decades of work when everything was fine or for work that person will do in the future. It is difficult … so, I think people refrain from saying anything” (W5).

Providers mentioned the concept of “the second victim syndrome” [30]: In addition to the primary victim of every medical error, there is a second victim, the physician who, as soon as he or she discovers a mistake, immediately feels guilty, and worried about what might happen in the future. These concerns might actually prevent physicians from sharing the mistake with the patient (W9).

Participants shared that engaging in disclosure has an emotional benefit, helping them cope with these difficult feelings: “…a sort of catharsis…you feel that you went back to interacting with the family, with the person” (W9);

So that I can continue to sleep at night, I'm not interested in the lawsuit... or what risk management will tell me... What I am interested in right now is my own healing in admitting my mistake, and this is a relief. The error really is no longer a secret I am trying to hide (W1).

Providers emphasized the importance of support for colleague in a vulnerable state after an error occurs: “To be there for him… to keep him away from the place, to let someone else take care of it …” (W7). “Perhaps on a voluntary basis, to ask a team member to provide emotional support and guidance…” (W7).

Theme 3: The medico-legal discourse challenges transparency.

Hospital directors, hospital risk managers, and risk department officials presented an ambivalent opinion towards transparency. On the one hand, they declared that transparency after the occurrence of an error is a fundamental part of the organizational commitment to improve patient care. They conveyed a reassuring message emphasizing their commitment to support physicians and nurses who report medical errors: “… we are not looking to punish anyone; we want to learn from the work processes, how we can improve the working environment and how we can avoid the next incident” (W4).

However, they expressed concern that the patients and their families might construe an apology as an admission of guilt and warned the providers to be careful. The risk managers emphasized that it is essential to report to them an error in medical treatment as soon as possible so that they can determine what had happened and plan an appropriate response. The hospital management did not deny that, in some cases, the investigation of the case could result in a penalty such as transferring someone from a particular ward: “… this idea that there’s a perfect world where we just empower people who made errors and surround them with empathy is just wrong” (W5).

Our observations revealed that the medical-legal discourse about transparency had an effect on the opinions and actions of health care providers discourse about transparency. They claim that the medico-legal atmosphere made it hard to learn from mistakes or to support the staff involved in the case:

I can investigate myself as much as I like, but I don’t leave any traces for outsiders. It hurts me because it prevents colleagues from learning from what happened. It is a lesson I learned the hard way... transparency is a good thing, but sometimes too much transparency is not … (W6).

Although they reiterated that transparency with patients is necessary, providers expressed uncertainty about how to disclose an error when one occurs without endangering themselves. They asked for practical tools:

Unfortunately, in our legal environment, we remain in a situation that an apology of a staff member may be viewed as an acknowledgment of responsibility, even if the intent was to express empathy and sorrow. Until this is changed, these words about transparency are nice, but they are empty (W9).

Theme 4: Providers and patients call for a change in the culture regarding disclosure of medical errors.

Providers and patients found a common language about their willingness to change “the day after” a medical error occurs.

Patients reported that the workshop restored their trust in the system. They were surprised at how much the physicians and nurses were willing to listen, to learn, and to improve. Patients were optimistic about the possible of a culture change within the medical system: **"**I’m optimistic because I’ve heard a few voices here say yes to transparency …I believe it’s a start of a change that will do us all good. It’s a win–win situation. We are all human. Good luck" (Patient, W9);

It was a pleasant surprise for me that the Ministry of Health pays attention to this issue. Thank you. I think there are people in management and on the medical team who want to work on it [disclosure] ... it’s amazing (Patient, W1).

Providers said that the patients’ stories strengthened their perception of the importance of listening to patients and trying to understand their feelings in order to avoid mistakes in the future. They were encouraged to tell the truth to the patients if a mistake has already occurred**:**

The patient is talking about providers’ education ... so physicians will not think that they are the right hand of God, but will listen to patients who know themselves and their own body. It is not just about mistakes. It is about how we treat our patients, how we listen to them, and how empathetic we are, and not how we deal with the shift, the load, and the daily chores (W1).

Providers discussed different ways disclosure can improve the culture of safety at the hospital. “I really believe in organizational culture … I think that forms are important…the so-called checklist… I’m standing in front of a mirror, in front of the list of things to check—got it, got it, got it, got it, excellent?” (W7); “I see the ‘near misses’ as a possibility for growth, which you report” (W2). Providers raised the importance of education to establish a culture of transparency: "We do not have the training to say we are sorry… we do not always know how to apologize properly. We must learn to debrief our mistakes for ourselves and the patient" (Medical team member to the patient) (W5).

Some suggested ways to implement transparency with patients in the daily routine of the hospital: “… At the end of the morning meeting, everyone says whether they made an error… and how they could have prevented it…change is possible” (W6). They discussed the changes that specific departments in their hospital had already implemented to encourage a culture of transparency in their organization, such as refreshing procedures for reducing the chance of errors.

Providers talked about recruiting experts from other professions — psychologists, social workers, professional conflict mediators, and others to help in carrying out transparency with patients and family and between the medical team members and the hospital’s management team. In one workshop, participants suggested integrating representatives from the risk management team into the hospital departments to facilitate their understanding of the daily dilemmas that providers face regarding transparency: “We want to have several people who speak the [legal] ‘language.’ who will lead us on this issue, who increase our vigilance and awareness …” (W3). In another workshop, participants suggested that one team member in the department can act as a liaison to update the patient or the relatives during the formal hospital inquiry.

Providers raised the importance of education to establish a culture of transparency: "We do not have the training to say we are sorry… we do not always know how to apologize properly. We must learn to debrief our mistakes for ourselves and the patient" (Medical team member to the patient (W5).

A few physicians mentioned the importance of nurturing critical thinking among interns and young nurses and teaching them how to give and accept feedback. “I think both nurses and doctors are sure they are excellent. There is an ego problem here. We are not open to receiving feedback…” (W12);

Modesty and doubt. … if we stuck to these two words when we’re learning, when we’re caring for patients, when we’re teaching, many of these problems would be resolved. It would be easier admit our errors, to apologize, to prevent them, and to move on because we would question everything (W7).

Some called for a deeper cultural change in the hospital team to reduce possible errors:

This is something that we need to consider in terms of the professional hierarchy. It appears that doctors are only allowed to be transparent with other doctors, nurses with nurses, but the rest of the hospital staff [secretaries, cleaners etc.], can sometimes prevent an error… but they are not part of the discussion …we’re all part of the same [work] place… and we all need to somehow fix this thing… (W4).

Few providers declared that they were starting an action: "We are about to set up a groupthink to try to understand this issue … Anyone interested in taking part in this? We will be happy to get help. We will try to build something that is both useful and right for the hospital" (W9).

### Survey

The responses to the survey distributed to the participants at workshops (see “Appendix”) suggested a general agreement with the principle of disclosure and benefit from the workshops.

## Discussion

There is little information about the disclosure of medical errors in Israel or the prevailing attitude of health care providers and administrators toward disclosure in general. This study summarizes research on 15 workshops conducted to encourage an open discussion on the disclosure of medical errors in healthcare settings, held in medical centers in Israel. These meetings provided a platform for a reflective discussion among various players and an opportunity for expressing emotions–in contrast to the adversarial disputes that frequently take place in the courtroom.

- Mutual listening, expressing emotions, and “transitional space”

In the workshops, participating patients told their stories to physicians and nurses whom they did not know at a hospital other than the one in which their case took place. Reflecting on our project, we recognize this as a key point. It may be that anonymity mitigated a potential emotional conflict and allowed what Martin Buber described as an “I-Thou” relationship [31], between the participants in the workshops in which one is receptive and open to being influenced by other beings and including oneself in the personal reality of the other [32].

Carl Rogers’ [33] “listening-with-understanding” approach draws attention to a listening activity intended to authentically achieve the other person’s reference point with respect and empathy [34]. As Gordon [32] expressed: “Listening plays an essential role in initiating many dialogues by creating a space in which two people can embrace each other as complete individuals”. Listening in an emphatic and non-judgmental way whereby the speakers feel the listeners accept them, rather than agree with them, reduces defensive reactions [35]. The recognition of each other’s feelings provided a common ground on which to base interaction between providers and the patient as partners [36]. While failure is often dealt with through cognitive responses, a focus on emotions can act as a motivator, allow for learning, and lead to increased effort to improve on past errors [37].

According to the patients’ responses at the final sessions, the setting had a healing effect and enabled them to be open to the concerns and difficulties of the medical team, thereby restoring their trust in the system. According to the providers’ responses the setting had gave them motivation to act for a change. We believe that the workshops provided a “transitional space” [38] for physicians, nurses and patients “… to free themselves, to a certain extent, from the shaping power of dominant fields…”, to challenge these dominant fields and to change [39]. The concept of transitional space captures the phenomenon of constructing new shared relationships, meanings, and rules of behavior through mutual interaction between people [40, 41] and raises the possible conditions for individual and collective growth [39].

- Responsibility, professional-moral obligation, and the medico-legal discourse

Overall, in our workshops there was agreement about the importance of disclosure. Physicians, in particular, discussed their professional responsibility and moral obligation for transparency with the injured patient and their families [14, 42]. They mentioned the value in learning from what happened in order to improve the quality and safety [5] of medical care.

The physicians and nurses who participated in our workshops were ambivalent about the consequences of taking responsibility or admitting guilt. They were worried that patients and families might use their honest apology against them [43]. However, transparency with patients and families after medical errors does not worsen liability outcomes. In fact, it is quite the opposite. Patients tend to turn to legal options not because of what occurred medically, but because of how they are treated when something unexpected occurs [44, 45]. When honest explanations and apologies are offered [46, 47], transparency facilitated reconciliation with the patient and support for involved caregivers. This is highly satisfying to patients and to clinicians [48]. LeCraw et al. [44] found that events resulting in injury due to medical error were resolved 43% of the time with apologies alone. Nevertheless, physicians infrequently offer complete apologies [49].

Collins et al. [50] found that physicians consider errors personal, not system failures and want to establish a blame-free culture. Cooper et al. [51] argue that it is difficult to promote an atmosphere of learning from medical errors without eliminating an atmosphere of blame. According to LaDonna et al. [52], physicians should practice strategies for coping with failure, while emphasizing the value of mentorship, self-care, and support. Bynum et al. [53] suggest developing approaches in medical education that enhance professionals’ resilience to medical errors, helping them acknowledge and confront shame, guilt, and pride. These approaches should also address providing effective feedback to colleagues without humiliating them, guiding learners to adopt shame-resilient approaches to errors and establishing the environmental conditions necessary for learners to willingly share emotions and seek help. For von Arx, et al. [37], the most important factor for members of a medical team after the occurrence of a medical error is receiving superiors’ support to reinforce their professional identity, thereby reducing job turnover.

In our workshops, the hospital administrators concurred with the idea of transparency. However, they voiced a medico-legal demand to report the case of a medical error, investigate, and, when necessary, discipline those involved and/or “deny and defend” to protect the institution. Little has been written about the challenge of combining such a disciplinary approach with empathic support to teams. Despite the atmosphere of reconciliation in our workshops, the challenge was and still is whether and how this contradiction can be resolved.

### Limitations

(1) Self-selected convenience sampling may reflect some inherent bias in the characteristics of participants (e.g., they may have had a personal, a professional, or another agenda). Such a bias implies that the sample might not be representative of the population studied or that an over-emphasis was placed on of some of the findings from the study. (2) Our analysis relied on what the participants expressed openly before their colleagues and supervisors. Each of them had personal interests and motives, and it is likely that there were thoughts, opinions and emotions that did not arise in the discussion. At the same time, we saw the importance of a diverse assembly of professions and officials, which resulted in a fruitful discussion from both an emotional and professional point of view. (3) Our original plan was to conduct a follow-up to learn about the long-term effect of participating in the workshops on the participants. However, our plans for follow-up interviews to substantiate our conclusions [26] were thwarted by the emergence of COVID-19. (4) Although the response rate to the surveys followings the workshops was lower than we had hoped, there appeared to be a sense of agreement on the principle of disclosure and the benefits of attending the workshops.

## Conclusions and implications for policy

The workshops described in this study may help foster a culture of institutional transparency following medical errors and set the stage for comprehensive interventions such as CRPs [13] to promote disclosure in a respectable, protective, and efficient way. Organizations can take steps to support patients and their families, including involving them in a research design and developing solutions and initiatives aimed at improving the quality of care [54, 55].

Nadler and Schnabel [56] indicate that reconciliation implies a process of learning in which greater trust occurs during social contacts. Furthermore, we believe that cooperative efforts aimed at common goals significant to both providers and patients and family members can create a process of reconciliation between patients and medical team members following a medical error.

The participants in our workshops shared their feelings about what is known as “the second victim syndrome” [30]. This refers to the feelings of the medical staff of guilt, loss of confidence, professional dissatisfaction, and burnout after making an error. A process of reconciliation should consider these feelings and respond adequately.

The COVID-19 pandemic disrupted our workshops. However, we believe that the burden on physicians and nurses and the danger of burnout has intensified since that time [57]. Measures such as listening and transparency, the challenges in communication between the providers, patients, and family members have assumed greater importance [58].

The current traumatic state of war in Israel following the horrendous and massive terror attack on October 7th has created major and unprecedented challenges, especially for healthcare and mental health. Without a prospect for recovery to routine work at this time, it seems appropriate to be cautious about proposing any strong policy recommendation these days.

Nevertheless, we suggest the following steps:

1. The Ministry of Health should resume the workshops described in this work for all hospitals and for all healthcare maintenance organizations.
2. In order to translate a transparency culture into practice, the Ministry of Health to should establish guidelines for healthcare teams with protocols determining who should communicate with patients and families after an error has occurred and how errors should be communicated to patients [54].
3. Regulatory bodies in charge of licensing and certification in the healthcare professions should make mandatory training for skills in disclosure as in a simulation-based workshop at the Israeli Center for Medical Simulation [22], as described by others [59].
4. Further resources, guidelines and practical tools for healthcare institutions and providers are available at the following link: http://bit.ly/honesty4msr.

## Data Availability

Not applicable.

## Appendix: Survey about attitudes towards disclosure of participants at workshops

An anonymous survey about attitudes towards disclosure was distributed to participants before beginning the workshop and its response rate was 24% (n = 239). The respondents agreed with the principle of disclosure after a medical error (97%), said that they behave according to the principle of transparency (92%) and claimed they provide an apology (77%—all percentages “to a great or very great extent”). Regarding others, they said that others behave according to the principle of transparency (51%), that the institutional culture allows transparency (75%), and that the executive management supports disclosure following a medical error (82%—all percentages “to a great or very great extent”).

An anonymous survey was also distributed to participants at the end of the workshop and its response rate was 10% (n = 101). The respondents said the workshop was interesting (84%) added new information (71%), raised awareness for the importance of transparency (75%) a sense of self-efficacy for disclosure (79%), and that it would be important for healthcare teams (88%—all percentages “to a great or very great extent”).

## Abbreviations

CRPs: Communication-and-resolution programs
Providers: Healthcare providers
